# Assessing the environmental sustainability of ethanol from integrated biorefineries

**DOI:** 10.1002/biot.201300246

**Published:** 2014-01-29

**Authors:** Temitope Falano, Harish K Jeswani, Adisa Azapagic

**Affiliations:** School of Chemical Engineering and Analytical Science, The University of Manchester Manchester, UK

**Keywords:** Biofuels, Environmental impacts, Ethanol, Integrated biorefineries, Life cycle assessment

## Abstract

This paper considers the life cycle environmental sustainability of ethanol produced in integrated biorefineries together with chemicals and energy. Four types of second-generation feedstocks are considered: wheat straw, forest residue, poplar, and miscanthus. Seven out of 11 environmental impacts from ethanol are negative, including greenhouse gas (GHG) emissions, when the system is credited for the co-products, indicating environmental savings. Ethanol from poplar is the best and straw the worst option for most impacts. Land use change from forest to miscanthus increases the GHG emissions several-fold. For poplar, the effect is opposite: converting grassland to forest reduces the emissions by three-fold. Compared to fossil and first-generation ethanol, ethanol from integrated biorefineries is more sustainable for most impacts, with the exception of wheat straw. Pure ethanol saves up to 87% of GHG emissions compared to petrol per MJ of fuel. However, for the current 5% ethanol–petrol blends, the savings are much smaller (<3%). Therefore, unless much higher blends become widespread, the contribution of ethanol from integrated biorefineries to the reduction of GHG emissions will be insignificant. Yet, higher ethanol blends would lead to an increase in some impacts, notably terrestrial and freshwater toxicity as well as eutrophication for some feedstocks.

## 1 Introduction

Despite many attempts worldwide to increase the share of renewable energy and so reduce our reliance on fossil fuels, with the market share of 87%, fossil fuels continue to be the major source of energy; by comparison, renewables contributes only 2% [1]. Current consumption of oil alone is around 88 million barrels per day [1] (∼15 million tonnes) and is predicted to increase by about 33% in the next 20 years [2]. In addition to the increasing pressure on finite reserves, the growing consumption of fossil fuels leads to a range of environmental impacts, including global warming, acidification, and ozone depletion. For example, fossil fuel combustion contributes 90% of the global CO_2_ emissions [3]. Thus, there is a clear need to explore alternative sources of fuels, including biofuels.

Biofuels are drawing increasing attention globally as they could help reduce the greenhouse gas (GHG) emissions and address energy security concerns in many countries. As a result, world ethanol production from biomass tripled between 2000 and 2007, from 17 billion to more than 52 billion litres, while biodiesel expanded 11-fold, from less than 1 billion to almost 11 billion litres [4]. Currently, the majority of the global biofuel production is ethanol from first-generation feedstocks, representing over 80% of liquid biofuels by energy content [4] with the USA, Brazil, and the EU being the main producers. Globally, investments into biofuel production capacity are in the region of $5 billion worldwide and are growing rapidly. Future estimates put the contribution of biofuels at 40–85 EJ/year by 2050; by comparison, current contribution from the fossil fuels totals 388 EJ/year [4].

However, the use of first-generation feedstocks such as corn and wheat for fuel production has become a contentious issue largely owing to competition with food production but also because of the GHG emissions associated with land use change (LUC) [5]. For these reasons, the focus is now shifting toward second-generation (lignocellulosic) feedstocks which could avoid food competition and LUC issues. These include energy crops (e.g. poplar, miscanthus, etc.) and wastes (e.g. agricultural, forestry, and municipal waste). To maximise their utilisation, it is envisaged that second-generation feedstocks will be used in integrated biorefineries to co-produce biofuels with chemicals and energy. Although there are currently no commercial facilities in operation, the principle of an integrated biorefinery is analogous to a conventional refinery, except that instead of fossil resources, they utilise different bio-feedstocks. An integrated biorefinery can use either a biochemical or thermochemical route, or a combination of both. Each route has its advantages and disadvantages but they all have to overcome a range of technological issues before they can become a commercial reality [6]. In addition to these, integrated biorefineries also face a number of sustainability challenges – environmental, economic, and social – which must be evaluated carefully on a life cycle basis to avoid shifting the issues along supply chains [7]. The life cycle approach is also required by various legislative acts related to biofuels, including the EU Renewable Energy Directive [8] and the US Energy Independence and Security Act [9].

This paper focuses on environmental sustainability of integrated biorefineries and specifically on the biochemical production route to assess life cycle impacts of ethanol co-produced with chemicals and energy from lignocellulosic feedstocks. While over 50 studies of environmental impacts of ethanol from lignocellulosic feedstocks have been carried out, more than half have considered only GHG emissions with a large number focusing on two feedstocks – corn stover and switchgrass – largely in the USA [10]. In this work, we go beyond previous studies to consider a range of environmental impacts and several different feedstocks, based on their availability around the world. The latter is discussed below, followed by an overview of conversion technologies envisaged for use in integrated biochemical refineries.

The main sources of lignocellulosic biomass include residues from agriculture and forestry as well as energy crops such as perennial grasses and different wood species. As shown in Supporting information, Table S1, the availability of different types of waste feedstocks varies by country and regions. For example, the main agricultural residues available in North America are corn stover and wheat straw [11]. The latter is also prevalent in Asia and Europe [12]. Currently, around 5.1 billion dry tonnes per year of agricultural residues, such as corn stover, wheat straw and rice husks, are produced globally [13]. Since they are less costly, annually renewable and widely available in many countries (Supporting information, Table S1), they have a huge potential to support and expand the biomass conversion industry in the long term. With yield and other technological improvements, it is expected that the availability in the future would increase significantly. For instance, in the United States, there is an estimated potential for producing 1.3 billion tonnes per year of agricultural and forestry waste without interfering with current land use; this would be enough to meet more than one-third of the current demand for transportation fuels [14]. In the United Kingdom, nearly 27 million tonnes of second-generation feedstocks could be available per year [15], of which agricultural and forestry residues are the most abundant, constituting more than 80% of the total potential. A further 10% could come from energy crops such as miscanthus and poplar. At present, the UK has the largest area under the energy crops in Europe. Cultivation in other European countries is also growing, including in Sweden, Finland, Germany, Spain, and Italy [16], as well as in North America [17].

Biochemical conversion of lignocellulosic biomass to fuels and chemicals typically involves pre-treatment, enzymatic hydrolysis, fermentation and product recovery. Pre-treatment, which can be physical, chemical or biological, helps in separating the cellulose and hemicellulose parts of lignocellulosic feedstock from the lignin [18]. During enzymatic hydrolysis, glycosidic bonds in the structure of cellulose and hemicellulose are converted by enzymes into sugar units such as glucose, xylose, etc. There are two types of enzymatic hydrolysis: separate hydrolysis and fermentation (SHF) and simultaneous saccharification and fermentation (SSF). The latter has some advantages over the SHF process such as better ethanol production rate, reduced product inhibitors formation, lower enzyme consumption, reduced volume of reactor and short residence times [19]. However, slow hydrolysis reaction and high cost of enzymes are the main limitations for both processes [20], although it is expected that costs will drop drastically with improvements in biotechnology and large scale production.

In the fermentation stage, glucose and xylose are converted to ethanol by fermenting micro-organisms such as bacteria, yeast, or fungi. Currently, the xylose fermentation technology is not as effective as glucose fermentation and further research is needed to develop xylose (pentose)-fermenting micro-organisms [21, 22] to increase the overall ethanol yield. Besides ethanol, several platform chemicals such as glycerol, succinic acid, and xylitol can be produced via fermentation [23]. Production of other chemicals such as butanol, acetone, ethyl acetate as well as acetic and lactic acids, would help to improve the profitability of biorefineries [20, 24]. Similar is true for the lignin and other solid residues which are removed from the fermentation broth by filtration and can be burned to recover energy. The commercial viability of biorefinery would be improved further through development of biotechnological processes for the production of polymers from lignin [25]; one such development is ongoing in Europe within the BIO-MIMETIC project [26].

## 2 Materials and methods

As mentioned earlier, there are no commercial integrated biorefineries in operation at present so that most studies are based on conceptual design. This study is based on the Aspen Plus model developed by NREL [27, 28]. However, since that considers corn stover as a feedstock, the ASPEN model has been modified for the purposes of this study to consider the following four feedstocks: wheat straw and forest residue as wastes and poplar and miscanthus as energy crops. These were chosen based on the global feedstock availability and future trends, as discussed in Section 1. Each feedstock is fed to the refinery one at a time rather than their blend so that four ASPEN models have been generated accordingly. The results from the ASPEN models have then been used to estimate the environmental impacts of ethanol production from different feedstocks. Life cycle assessment (LCA) has been used as a tool for these purposes, following the guidelines in the ISO 14040 and 14044 LCA standards [29, 30]. The environmental impacts have been estimated using the LCA software GaBi v.4.4 [31] according to the CML 2 Baseline 2001 methodology [32]. The following environmental impacts are considered: abiotic depletion, acidification, eutrophication, freshwater, marine and terrestrial ecotoxicity, human toxicity, global warming, ozone layer depletion, photochemical smog, and land use.

The next sections describe the goal and scope of the study, the system considered and the assumptions. Further details can be found in Supporting information.

### 2.1 Goal and scope of the study

The main objective of this study is to assess and compare the environmental sustainability of ethanol produced in an integrated biochemical refinery using different second-generation feedstocks. A further goal is to find out how environmentally sustainable such ethanol is compared to first-generation ethanol as well as ethanol and petrol produced from fossil resources in conventional refineries. The study aims to inform both industry and policy makers as well as consumers.

**Figure 1:**
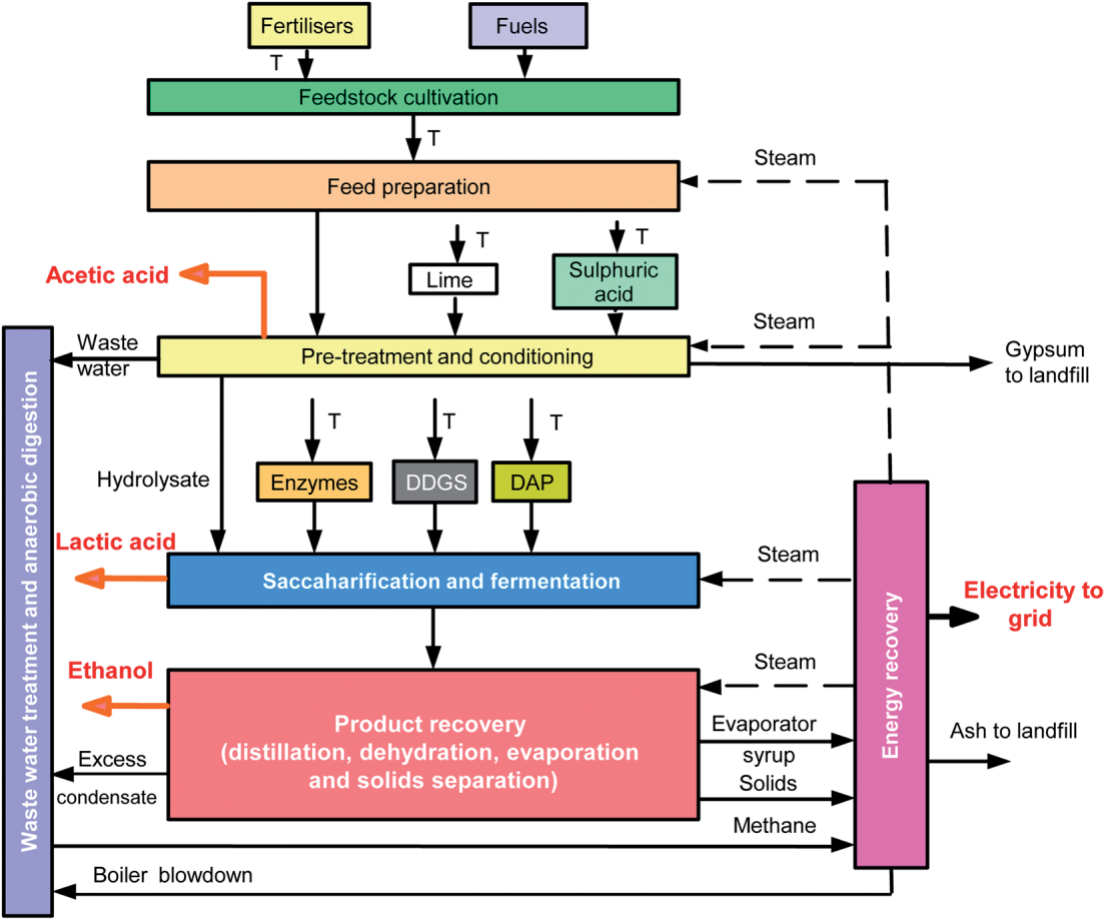
System boundaries for the integrated biorefinery from “cradle to gate.” The system boundary comprises feedstock cultivation (where relevant), collection and transportation to the refinery and production of ethanol, acetic and lactic acids, electricity, and heat.

As shown in Fig. 1, the system boundaries are from “cradle to refinery gate” and include feedstock cultivation (where relevant), collection, and transportation to the refinery, and production of ethanol and its co-products, here assumed to be acetic and lactic acids and electricity. Therefore, the distribution and use of the products are excluded from this study. However, the use of ethanol is considered in the latter parts of the paper when ethanol is compared with petrol, so that the system boundary then becomes from “cradle to grave” for both. The impacts from construction and decommissioning of the refinery are also excluded from the study as the contribution of infrastructure to the impacts of products is typically negligible owing to the long lifetimes of industrial installations.

The functional unit is defined as the production of 1 litre of ethanol. To enable comparisons of ethanol from different feedstocks, the amount of ethanol produced in the refinery is assumed to be equal for each feedstock; instead, the respective amounts of the feedstocks and the output of the co-products vary, depending on the composition of the feedstock. For the former, see Supporting information, Table S2 and the latter Supporting information, Table S3. The study is assumed to be based in the United Kingdom. More detail on the system is given below.

### 2.2 System description

The refinery comprises the following main units: feedstock handling and pre-treatment, saccharification and fermentation, product and energy recovery, and wastewater treatment (Fig. 1). These are described briefly next; the process conditions are equivalent to those in the NREL design [27, 28].

#### 2.2.1 Feedstock preparation and pre-treatment

After harvest or collection, as relevant, the feedstocks are transported to the refinery by trucks, assuming a 100 km distance. The amount of ethanol recovered from different feedstocks depends on their cellulose content; for example, as shown in Supporting information, Table S3, forest residue has the highest and wheat straw the lowest cellulose content. Given that the amount of ethanol produced from each feedstock is fixed to allow comparisons between the feedstocks, the quantity of each feedstock needed to produce this amount of ethanol is estimated based on the cellulose content and this is reflected in Supporting information, Table S2.

Once in the refinery, the feedstock is cut, washed, and shredded, ready for the pre-treatment process. Here, the feedstock is treated with dilute sulphuric acid at elevated temperature (190°C) to dissolve the hemicellulose to soluble sugars, namely xylose, mannose, arabinose, and galactose. Supporting information, Table S4 shows the hydrolysis reactions of the hemicellulose and the fraction converted to products. The acid hydrolysis also librates inhibitors such as acetic acid which can be toxic to the microorganisms in the fermentation stage but is also a commercial product; therefore, acetic acid is separated at this stage. After the pre-treatment, the resulting material is flash cooled, washed and filtered to separate the liquid portion of the hydrolysate from the solids (cellulose). The liquid portion is then treated with lime and pH readjusted by the addition of sulphuric acid. This leads to the formation of gypsum, which is filtered and after further treatment can be sold as a commercial product. However, in this study, it is assumed that gypsum is landfilled as waste (Fig. 1). Finally, the treated liquid hydrolysate is remixed with the solid portion and the resulting slurry sent to the saccharification and fermentation stage.

#### 2.2.2 Saccharification and fermentation

In this stage, cellulase enzymes are used to assist the saccharification of cellulose to glucose. These include endoglucanases for polymer size alteration, exoglucanases for crystalline cellulose hydrolysis and B-glucosidase for cellobiose hydrolysis to glucose [27]. The resulting glucose and xylose are then fermented to ethanol by *Zymomonas mobilis*. Dried distillers' grains with solubles (DDGS) and diammonium phosphate (DAP) are added as nitrogen sources required for *Z. mobilis'* growth. *Escherichia coli* is also utilised at this stage to produce lactic acid from glucose and xylose [33]. Supporting information, Table S5 summarises the reactions taking place at this stage.

#### 2.2.3 Product recovery

Ethanol recovery from the fermentation beer is accomplished via a two-column distillation and molecular sieve adsorption. In the first column, the feed is pre-heated with flash vapours from the pre-treatment unit and further heated through exchange with the bottoms from the first distillation column. This removes any CO_2_ and about 90% of water to recover ethanol which is then fed to the second column where it is concentrated to a near azeotropic composition. The water from this mixture is then removed by vapour phase molecular sieve to produce 99.5% pure ethanol. Bottoms from the first distillation containing unconverted insoluble materials are dewatered by a pressure filter and sent to the boiler for energy recovery. The liquid from the filter that is not recycled back to the process is concentrated in a two-stage evaporator using waste heat from the distillation. The resulting concentrated syrup is sent to the energy recovery unit.

#### 2.2.4 Wastewater treatment

The on-site plant treats wastewater from different parts of the refinery, including various condensates and boiler blowdown. A combination of aerobic and anaerobic treatment is used for these purposes. The treated water is recycled back in the process while the activated sludge from the aerobic and methane from the anaerobic treatment are burned to recover energy.

#### 2.2.5 Energy recovery

Various waste streams are burned in the combustor to produce electricity and steam for the process as well as export the excess of electricity for sale. In addition to the methane and activated sludge from wastewater treatment, these include residual lignin and concentrated syrup from the evaporator. The boiler produces steam which is fed to a multistage turbine to generate electricity. Steam is also extracted at various conditions and used as process heat (Supporting information, Table S2). It is assumed that 15% of the heat content of the feed into the combustor is converted to electricity and 51% to steam [27]. It is estimated that the plant consumes 96480 MWh/year of electricity; the excess is sold to the grid (see Supporting information, Table S2). The ash from the combustor is landfilled.

### 2.3 Data sources

As mentioned previously, there are no commercial integrated biorefineries yet, so that the data for the biorefinery are based on a conceptual design proposed by NREL and modelled in Aspen Plus [27, 28]. As also mentioned earlier, this model has been adapted to consider different feedstocks. All other background data are from the LCA database Ecoinvent [34], except for poplar and miscanthus which are from GEMIS [35] as these were not available in Ecoinvent. The LCA data for enzymes were not available in any of the LCA databases but data for the GHG emissions have been found in literature [36] and used here; therefore, the contribution of enzymes to the other impacts is excluded. The LCA data for transport are also from the Ecoinvent database, assuming that all the materials used in the system are transported for 100 km by a 40 t truck.

### 2.4 Allocation of environmental impacts

Since an integrated biorefinery is a multi-output system, the method for allocating the environmental impacts between the co-products is important as it can affect the results. Following the ISO 14040/44 guidelines [29, 30], “system expansion” has been used and the system has been credited for producing the co-products in alternative production systems. The system is credited only for the products that leave the system to be sold – i.e. the acids and electricity. Therefore, no credits are given for the heat as all of it is used by the refinery; however, the system benefits from not using fossil fuels to generate heat. For the credits, acetic acid is assumed to be produced from butane and electricity from the UK grid and their impacts have been subtracted from the total impacts from the system. The LCA data for the credits have been sourced from the Ecoinvent database [34]. Owing to a lack of specific LCA data for lactic acid production, generic data have been used corresponding to the average impacts from different organic chemicals, again sourced from Ecoinvent. While this means that the results for lactic acid may be either over or underestimated, because of its relatively low amount (see Supporting information, Table S2), the effect on the results may not be significant.

Furthermore, to gauge the effect of allocation on the results, economic allocation has also been performed. For this, the impacts have been allocated between ethanol and its co-products in proportion to their respective market prices shown in Supporting information, Table S6.

## 3 Results and discussion

This section presents and discusses the results first for system expansion and then for economic allocation.

### 3.1 System expansion

**Figure 2:**
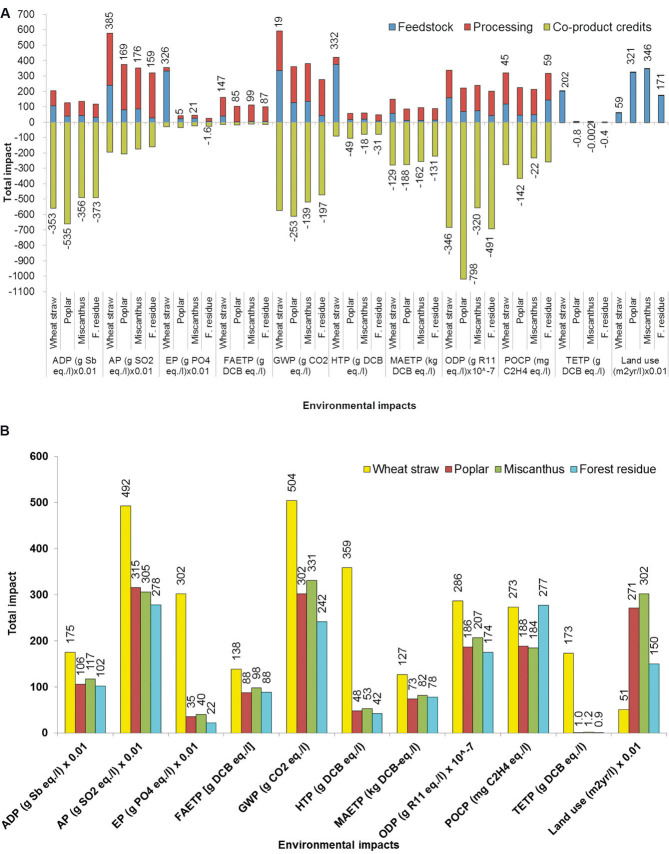
Life cycle environmental impacts of 1 litre of ethanol produced in integrated biorefinery. (A) The results shown for system expansion, whereby the system is credited for production of acetic and lactic acids and electricity. The values shown against each bar in the figure indicate the total impact taking into account co-product credits. (B) The results shown for economic allocation where the impacts are allocated to the co-products in proportion to their economic value. System boundary for both figures: cradle to gate. To obtain the original values of the impacts, multiply the values shown on the graphs by the multiplication factor shown on the x-axis for relevant impacts.

These results are shown in Fig. 2A and are discussed for each impact in turn below. As can be seen, seven out of 11 environmental impacts from ethanol are negative, including GHG emissions, indicating environmental savings. Ethanol from poplar is the best option for eight out of 11 impacts considered, with forest residue having the lowest acidification and eutrophication potentials and wheat straw requiring the least land area. Ethanol from wheat straw is the worst option for most impacts. The exceptions to this are ozone layer depletion and land use, for which miscanthus has the highest impacts, as well as photochemical smog for which forest residue is the worst option.

#### 3.1.1 Abiotic depletion potential (ADP)

This impact is negative for all four feedstock options, indicating a saving in abiotic resources (fossil fuels and elements). The ethanol from poplar is the best option with ADP of –5.35 g Sb eq./L ethanol. This is mainly due to the higher amount of the acetic acid produced (because of the higher acetate content) and the associated credits for its production. Wheat straw is the worst option but it still saves 3.53 g Sb eq./L ethanol. For ethanol from wheat straw, the impact from the feedstock and processing is roughly equal while for the other three options processing contributes around 70% to the total (this refers to the impacts before the credits for the co-products).

#### 3.1.2 Acidification potential (AP)

Ethanol from wheat straw has the highest AP (3.85 g SO_2_ eq./L ethanol) and from forest residue the lowest (1.59 g). This is despite the fact that the refinery using wheat straw generates the largest amount of electricity as the related credits are counterbalanced by the impact from agriculture which contributes around 40% to the total impact (before credits). By comparison, the contribution from this stage for the other feedstocks is around 20%.

#### 3.1.3 Eutrophication potential (EP)

Ethanol from wheat straw also has the highest EP, estimated at 3.26 g PO_4_ eq./L ethanol because of nutrients emitted in the agricultural stage which contribute over 90% to this impact. The lowest EP is for forest residue, which is negative indicating a saving of 0.02 g PO_4_ eq./L ethanol. This is due to both low impact from agriculture but also the credits for electricity and acetic acid.

#### 3.1.4 Freshwater ecotoxicity potential (FAETP)

The same pattern is noticed for this impact as for the others above, with poplar being the best option at 85 g DCB eq./L and wheat straw the worst with 147 g. Over 97% of this impact is from the production process for all the feedstocks except for wheat straw, for which production contributes 75%. This is mainly due to the disposal of ash and related emissions of heavy metals (nickel, copper, zinc, and vanadium).

#### 3.1.5 Global warming potential (GWP)

The GWP per litre of ethanol ranges from –253 g CO_2_ eq./L ethanol for poplar to 19 g for wheat straw. As for the previous two impacts for wheat straw, the GHG emissions from agriculture are higher than the credits for the avoided impact from electricity generation. For the other feedstocks, the production stage contributes most to the GHG emissions, and particularly enzymes, DAP and DDGS. Note that the GWP refers to the fossil carbon – the biogenic carbon storage is not considered as this carbon is released during the use of ethanol in vehicles (considered later in the paper).

#### 3.1.6 Human toxicity potential (HTP)

This impact is negative for three of the feedstocks: poplar (which is the best option at –49 g DCB eq./L), forest residue (–31 g), and miscanthus (–18 g). These savings are largely due to the credits for electricity. The highest value is found again for the straw amounting to 332 g DCB eq., largely (88%) from fertilisers and pesticides used for cultivation.

#### 3.1.7 Marine aquatic ecotoxicity potential (MAETP)

The MAETP is also negative across all the feedstocks, with ethanol from poplar having the greatest savings (–188 kg DCB eq./L) and wheat straw and forest residue the lowest (–129 and –131 kg, respectively). These savings are again largely (60%) due to the credit for electricity generation. The majority of the impact before the system credits is from processing and in particular from the ash disposal and materials used for pre-treatment and fermentation.

#### 3.1.8 Ozone layer depletion potential (ODP)

This impact is also negative for all the feedstocks, ranging from –79.8 μg R11 eq./L for poplar to –32 μg for miscanthus. The majority (>50%) of the ODP before system credits is from the life cycles of lime and DDGS.

#### 3.1.9 Photochemical oxidant creation potential (POCP)

Also known as photochemical smog, the POCP is lowest for poplar (–142 mg C_2_H_4_ eq./L) and highest for forest residue (59 mg) and wheat straw (45 mg), respectively. The impact from miscanthus ethanol is also negative (–22 mg C_2_H_4_ eq./L. The majority of the POCP is from the process (>55%) largely due to the emissions of carbon monoxide and nitrogen oxides from the combustor.

#### 3.1.10 Terrestrial ecotoxicity potential (TETP)

Ethanol from wheat straw has the highest TETP, estimated at 202 g DCB eq./L ethanol. This is almost entirely due to the impact associated with the feedstock production and in particular heavy metals in straw [34]. For all other feedstocks, there are small savings in this impact (0.002–0.8 g). The majority (>60%) of the TETP for these feedstocks is from the processing stage and particularly from the life cycle of sulphuric acid.

#### 3.1.11 Land use

At 0.59 m^2^ year/L of ethanol, wheat straw is the best option while miscanthus has the highest land requirement of 3.46 m^2^ year/L. This is almost entirely due to the land required for the feedstock, with the refinery contributing less than 1%.

### 3.2 Economic allocation

If economic allocation is used instead of system expansion, around 85% of each impact is allocated to ethanol, 6% each to electricity and acetic acid and the rest to the lactic acid; this is because of their respective quantities and market prices (see Supporting information, Tables S2 and S6). The results in Fig. 2B indicate a similar trend as for the system expansion except that now ethanol from forest residue is overall the best option, followed closely by poplar. An exception to this is photochemical smog for which forest residue is the worst option and miscanthus the best. Wheat straw is worst for all impacts except for land use for which it is best. Therefore, while the absolute results are quite different between the two allocation methods, the ranking of the options remains roughly the same across the impacts.

### 3.3 Land use change (LUC)

As mentioned in the introduction, LUC can lead to significant GHG emissions. Therefore, this section considers the effect of possible direct LUC on the GWP of ethanol; consideration of indirect LUC is outside the scope of this paper. LUC is relevant to energy crops; therefore, only poplar and miscanthus are considered. To cover different possibilities with respect to the current land use using the UK as an example, the following is explored:

(i) *Poplar:*

• conversion of current forest land to poplar forest assuming the GHG emissions of 2.5 t CO_2_ eq./ha·year [37]; and

• conversion from grassland to forest with –1.5t CO_2_ eq./ha·year (the negative value is due to the GHG emissions from grassland clearing being lower than the sequestration by poplar forest [37]).

(ii) *Miscanthus:*

• conversion from forest land to perennials with 20t CO_2_ eq./ha·year [38]; and

• conversion of grassland to perennials with 6.7t CO_2_ eq./ha·year [38].

**Figure 3:**
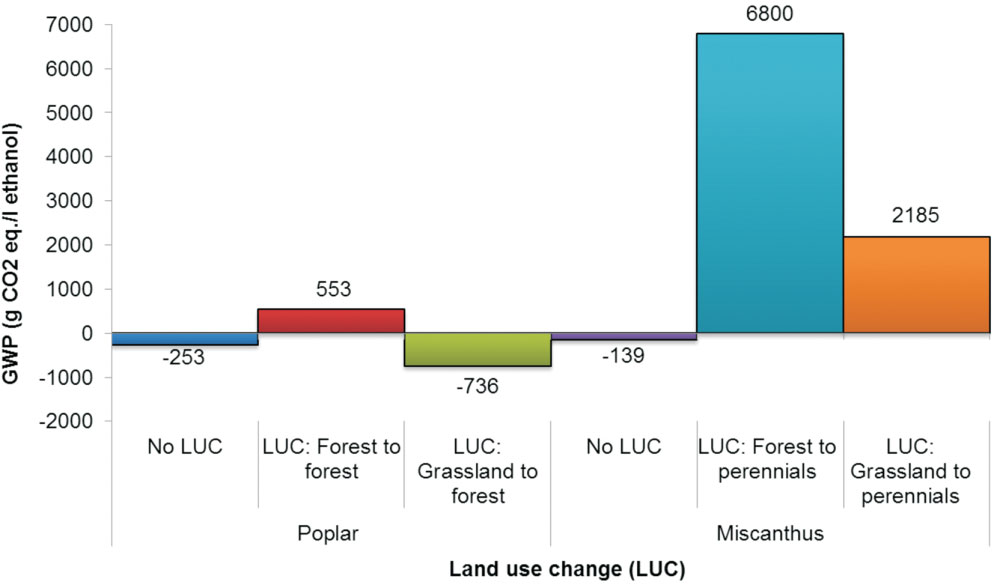
Impact of land use change on the global warming potential of ethanol. Results shown for system expansion with the system credited for co-producing acetic and lactic acids and electricity. System boundary: cradle to gate.

Figure 3 shows the potential effect of direct LUC on the GWP of ethanol. When current forest is converted to poplar forest, the GWP increases from –253 to 553g CO_2_ eq./L ethanol. However, when grassland is converted to poplar forest, the GWP is actually reduced to –736 g CO_2_ eq. because carbon sequestration by the forest is higher than the emissions released during the grassland conversion. For miscanthus, the effect of LUC is more dramatic: conversion of forest land increases the impact from –139 g CO_2_ eq./L to 6800 and to 2185 g for grassland conversion. Therefore, the results are very sensitive to the LUC and this aspect must be taken into account with any future production of ethanol from energy crops in integrated biochemical refineries since in some cases the GWP savings from the co-product credits are not enough to compensate for the emissions associated with the LUC. It should be noted that the effect of LUC would be much more pronounced if ethanol alone was produced in the refinery without the other co-products as there would be no credits for their production to compensate for the carbon emissions released from LUC.

### 3.4 Comparison with ethanol from fossil-based resources

This section compares the environmental impacts of ethanol produced in an integrated biochemical refinery with the impacts of ethanol produced in a conventional refinery from fossil resources, specifically from ethylene. The LCA data for the latter are from the Ecoinvent database [34].

**Figure 4:**
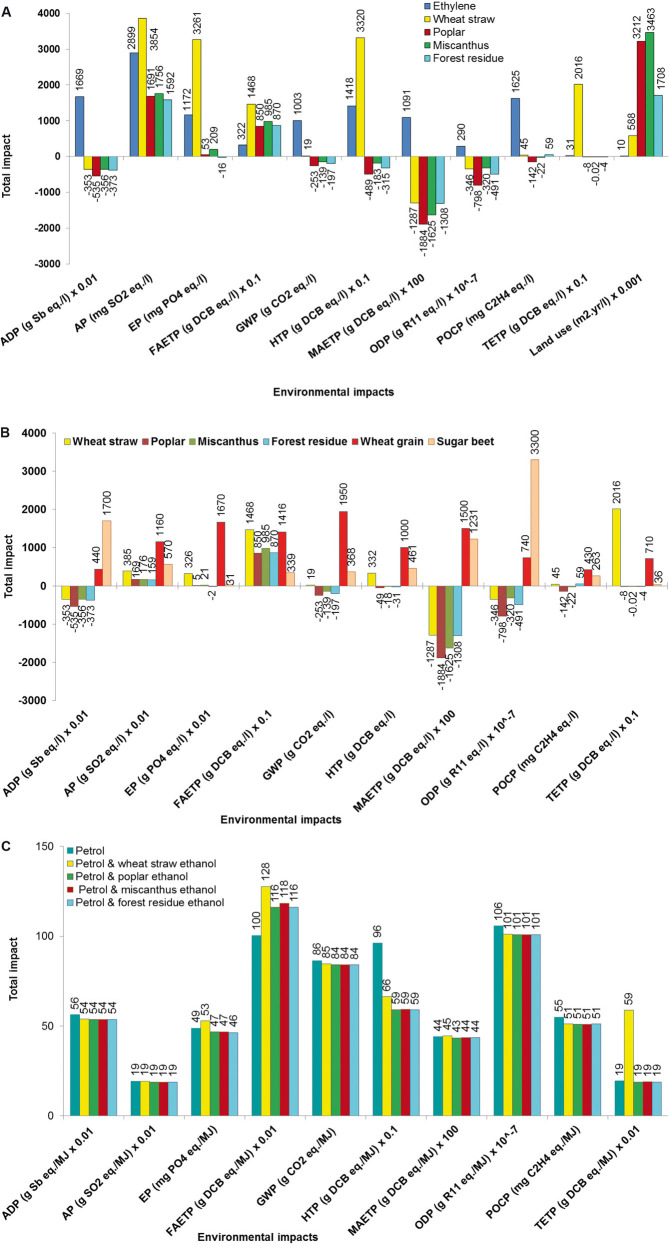
Comparison of ethanol from integrated biorefinery with other sources of ethanol and with petrol. (A) Environmental impacts comparison with ethanol produced from ethylene. Impacts shown for system expansion. System boundary: cradle to gate. (B) Environmental impacts comparison with ethanol from first-generation feedstocks (wheat and sugar beet). Impacts shown for system expansion. System boundary: cradle to gate. Results for land use not available for wheat and sugar beet ethanol and so this impact is not shown. (C) Environmental impacts comparison of ethanol/petrol mixture (5 vol%/95 vol%) with petrol. Results shown for economic allocation. System boundary: cradle to grave. To obtain the original values of the impacts in all figures, multiply the values shown on the graph by the multiplication factor shown on the x-axis for relevant impacts.

The comparisons can be seen in Fig. 4A. Compared to ethanol from poplar, miscanthus, and forest residue, ethanol from ethylene has higher impacts for most impacts, except for freshwater aquatic toxicity and land use. However, compared to wheat straw, it is a better option for six out of 11 impacts: acidification, eutrophication, freshwater, terrestrial and human toxicity, and land use. Therefore, while ethanol from second-generation feedstocks is a better option for some impacts, notably depletion of fossil resources and GWP, some of its other impacts are higher than for ethanol from fossil-feedstocks so that some trade offs are necessary. This is often ignored in current debates on biofuels, where the focus is largely on GHG emissions alone.

### 3.5 Comparison with ethanol from first-generation feedstocks

It is also important to compare the impacts of ethanol from second- to those from first-generation feedstocks. As an illustration, wheat and sugar beet cultivated in the United Kingdom are considered here. The data for ethanol from wheat grain and sugar beet are taken from CCaLC [39] and Foteinis et al. [40], respectively, as these data were not available in Ecoinvent.

As can be seen from Fig. 4B, ethanol from the second-generation feedstocks is environmentally more sustainable for most impacts than ethanol from the first-generation feedstocks considered here. The exceptions are eutrophication for which ethanol from sugar beet is a better option than from wheat straw as well as freshwater and terrestrial ecotoxicity for which ethanol from both wheat and sugar beet has lower impacts than from the straw.

### 3.6 Comparison with petrol

In this section, the impacts of ethanol from the second-generation feedstocks considered in this paper are compared to petrol. Here, the comparison is carried out from “cradle to grave” to account for the impacts during the use of fuels. This is particularly important for the GWP because of the emissions of fossil CO_2_ from petrol. To account for a different energy content in ethanol and petrol and enable fair comparisons, their impacts are compared per MJ. For these purposes, the impacts of ethanol per litre presented above have been converted assuming a lower heating value of 21.5 MJ/L. The life cycle of petrol comprises crude oil extraction and processing to produce petrol and its use in vehicles; the LCA data are from Ecoinvent [34]. The LCA data for the use of ethanol in vehicles are also from Ecoinvent and they have been added to the “cradle to gate” impacts estimated here.

**Figure 5:**
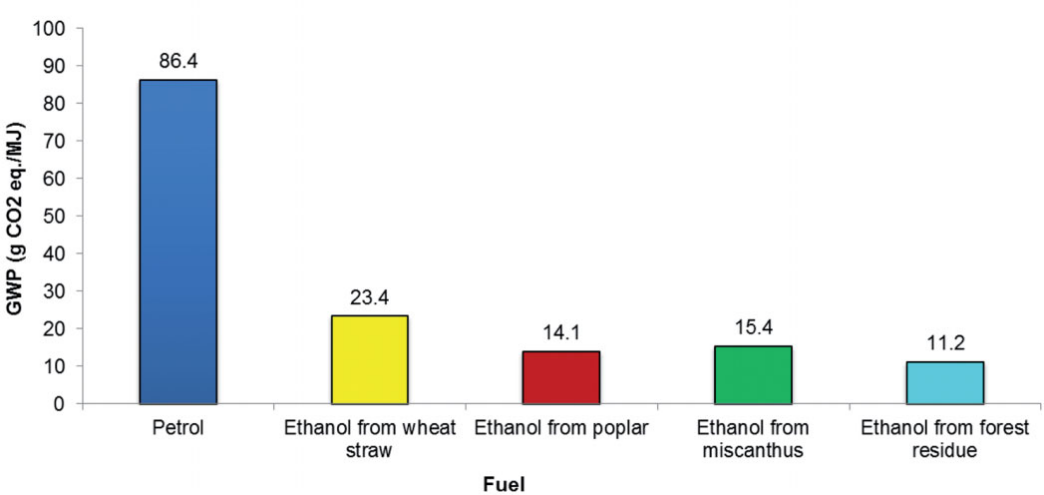
Comparison of the global warming potential of ethanol with petrol from “cradle to grave.” Results shown for economic allocation. Petrol: low sulphur.

The results for the GWP are shown in Fig. 5 indicating that there are significant GHG emissions savings from ethanol compared to petrol, ranging from 72% for ethanol from wheat straw to 87% for forest residue. However, this is on the basis of a 100% replacement of petrol by ethanol, which is currently not practiced almost anywhere in the world (except in, e.g. Brazil). In the United Kingdom, for instance, the British Standard for unleaded petrol allows only up to 5% ethanol as a blend component [41].

Therefore, assuming 5 vol% in petrol would lead to a 2–2.8% saving of GHG emissions per MJ of fuel (see Fig. 4C). To put this in perspective, GHG emissions from vehicles in the UK were 68 M t CO_2_ eq. in 2010 representing 12% of all national GHG emissions in the same year [42]. If all petrol were to be mixed with 5% of the types of ethanol considered here, the annual reduction in GHG emissions would be on average 0.3%, amounting to a total reduction of around 10% by 2050. Arguably, this is a small saving overall and far from the ambitious UK target of 80% reduction of GHG emissions by then [43]. Therefore, unless much higher amounts of ethanol were added to petrol, the benefits in terms of GHG emissions would be small. Similar is true for the other environmental impacts (Fig. 4C) for which the savings relative to pure petrol range from 2 to 4%. The exception to this is human toxicity for which much larger savings are observed – between 31 and 39%, depending on the feedstock.

On the other hand, some of the impacts from the blend are higher for ethanol from wheat straw than that for pure petrol. These are terrestrial toxicity which is three times higher, freshwater ecotoxicity which is 27% higher and eutrophication which is 8% higher. Freshwater ecotoxicity is also 13–15% higher than for petrol for all the feedstocks apart from miscanthus.

Therefore, for the current blends of ethanol and petrol for use in vehicles, the savings in GHG emission and some other impacts are small. However, higher ethanol blends would lead to an increase in some other impacts, notably terrestrial and freshwater toxicity as well as eutrophication for some of the feedstocks.

## 4 Concluding remarks

This paper has considered the life cycle impacts of ethanol produced in a biochemical refinery which also co-produces acetic and lactic acids and electricity. Four different feedstocks have been considered: wheat straw, poplar, miscanthus, and forest residue. The results suggest that when the system is credited for the avoided impacts for the co-products, seven out of 11 impacts are negative, suggesting environmental savings. These include depletion of abiotic resources and GHG emissions. Of the four feedstocks considered, ethanol from poplar is the best option for eight impacts. For the remaining three, forest residue has the lowest acidification and eutrophication potentials and wheat straw requires the least land. Ethanol from wheat straw is the worst option for most other impacts. The exceptions to this are ozone layer depletion and land use, for which miscanthus has the highest impacts, and photochemical smog for which forest residue is the worst option.

When economic allocation is used to apportion the impacts among the co-products using their market value, ethanol from forest residue becomes the best option across most impacts, but is followed closely by poplar. An exception to this is photochemical smog for which forest residue is the worst option and miscanthus the best. Wheat straw is the worst option for all impacts except for land use for which it is the best option.

LUC has a much more pronounced effect on the results than the allocation method. For miscanthus, conversion of forest land to perennial land increases the impact from –139 g CO_2_ eq./L to 6800 and to 2185 g for conversion of grassland. In the case of poplar, if another type of forest is converted to a poplar forest, the GWP increases from –253 to 553 g CO_2_ eq./L ethanol. However, when grassland is converted to poplar forest, the GWP is reduced to –736 g CO_2_ eq. because of the carbon sequestration by the forest. Therefore, the results are very sensitive to the LUC and this aspect must be taken into account with any future production of ethanol from energy crops in integrated biochemical refineries (or elsewhere).

Compared to poplar, miscanthus, and forest residue, ethanol from fossil feedstocks such as ethylene has higher impacts for most impacts, except for freshwater aquatic toxicity and land use. However, compared to wheat straw, it is a better option for six out of 11 impacts: acidification, eutrophication, freshwater, terrestrial and human toxicity, and land use. Therefore, while ethanol from second-generation feedstocks is a better option for some impacts, it is not necessarily environmentally more sustainable than fossil-derived ethanol for all the impacts.

However, compared to first-generation, second-generation ethanol is more sustainable for most impacts. The exceptions are eutrophication for which ethanol from sugar beet is a better option than from wheat straw as well as freshwater ecotoxicity and terrestrial ecotoxicity whereby both wheat and sugar beet have lower impacts than the straw.

Finally, in comparison to petrol, there are significant GHG emissions savings from ethanol, ranging from 72% for wheat straw to 87% for forest residue. However, this is on the basis of a 100% replacement of petrol by ethanol. Taking into account the currently prevalent 5% ethanol blends, the savings are much smaller, up to 2.8%. If 5% ethanol were to be added to all petrol used today in the UK, the GHG emission saving at the national level would be only 0.3% per year or around 10% by 2050. Similar is true for the other impacts except for human toxicity where the savings are potentially larger. Therefore, unless higher ethanol blends were used, the contribution of second-generation ethanol to the reduction of GHG emissions would be insignificant.

## Abbreviations

ADP: abiotic depletion potential
AP: acidification potential
DAP: diammonium phosphate
DDGS: dried distillers' grains with solubles
EP: eutrophication potential
FAETP: freshwater aquatic ecotoxicity potential
GHG: greenhouse gas
GWP: global warming potential
HTP: human toxicity potential
LUC: land use change
MAETP: marine aquatic ecotoxicity potential
ODP: ozone depletion potential
POCP: photochemical ozone creation potential
TETP: terrestrial ecotoxicity potential

## Supporting Information

As a service to our authors and readers, this journal provides supporting information supplied by the authors. Such materials are peer reviewed and may be re-organized for online delivery, but are not copy-edited or typeset. Technical support issues arising from supporting information (other than missing files) should be addressed to the authors.

suppinfo
