# Supplementary material for: Assessing the environmental sustainability of ethanol from integrated biorefineries
Source: Biotechnol J. 2014 Jan 29;9(6):753–65. doi: 10.1002/biot.201300246 (PMC4674963; doi:10.1002/biot.201300246)
Supplement: Supplementary file 1 — suppinfo [file biot0009-0753-sd1.pdf]

Biotechnology
Journal

Supporting Information for DOI 10.1002/biot.201300246

Assessing the environmental sustainability of ethanol from integrated biorefineries

Temitope Falano, Harish K. Jeswani and Adisa Azapagic

© 2014 Wiley-VCH Verlag GmbH & Co. KGaA, Weinheim

Assessing the environmental sustainability of ethanol from integrated biorefineries

Temitope Falano, Harish K. Jeswani and Adisa Azapagic

Supplementary information

Table S1 Global availability of agricultural and forest residue

Feedstock		Estimated global production (million dry tonnes per year)	Major producer countries
Agricultural residue [1]	Corn stover	1,413	USA, China, Brazil, Argentina, Ukraine
	Rice straw	1,084	China, India, Indonesia Bangladesh, Vietnam
	Wheat straw	1,056	China, India, Russian Federation, USA, France
	Sugarcane bagasse	502	Brazil, India, China, Thailand, Pakistan
Forest residue [2]		501	Canada, China, USA, Brazil, Sweden

Table S2 Summary of inputs into and outputs from the bio-refinery

	Wheat straw (t/yr)	Poplar (t/yr)	Miscanthus (t/yr)	Forest residue (t/yr)
**Inputs**				
Biomass (wet)	908,263	779,880	836,160	795,960
Water	1,918,593	1,647,404	1,766,292	1,681,373
Enzymes	63,219	54,278	58,194	55,396
Lime	22,182	19,047	20,422	19,441
Sulphuric acid	30,423	26,130	28,011	26,669
Dried distillers' grains with solubles (DDGS)	12,092	10,388	11,135	10,605
Diammonium phosphate (DAP)	1,520	1,302	1,399	1,335
Electricity (MWh/yr)[a]	96,480	96,480	96,480	96,480
Steam[a]	496,550	426,362	457,131	435,154
**Outputs**				
Ethanol	192,960	192,960	192,960	192,960
Acetic acid	25,575	41,358	20,285	27,256
Lactic acid	3,184	2,774	2,846	2,525
Electricity (MWh/yr)	171,192	156,333	160,800	129,400
Gypsum	66,845	57,398	61,538	52,662
Ash	40,200	33,768	36,180	32,160

[a] Generated by the refinery

Table S3 Feedstock composition

Feedstock composition (%)	Wheat straw [3]	Poplar [4, 5]	Miscanthus [6]	Forest residue [7]
Cellulose	32.6	42.7	38.2	44.1
Xylan	19.2	19.1	19.0	9.3
Arabinan	2.4	0.8	1.8	1.5
Galactan	0.8	0.2	0.4	2.0
Mannan	0.3	3.9	3.1	8.6
Lignin	16.8	27.7	25.0	27.4
Ash	10.2	1.0	2.0	0.9
Extractives	12.9	0.0	6.9	3.4
Acetate	2.2	4.6	1.8	2.8
Moisture content	15.0	50.0	15.0	70.0
Ultimate analysis				
C	43.9	50.9	48.1	52.7
H	5.3	6.0	5.4	5.4
O	38.7	41.9	42.2	41.1
N	0.6	0.2	0.5	0.7
S	0.2	0.1	0.1	0.1
LHV (MJ/kg)	17.6	18.7	17.2	16.4

Table S4 Pre-treatment reactions and assumed conversion rates

Reaction	Fraction converted to product
Xylan + nH_20 → Xylose	0.85
Arabinan + nH_20 → Arabinose	0.75
Galactan + nH_20 → Galactose	0.75
Mannan + nH_20 → Mannose	0.75
Acetate → Acetic acid	1

Table S5 Fermentation reactions and assumed conversion rates

Reaction	Fraction converted to product
Cellulose + H_2O → 2 Glucose	1
Glucose → 2 Ethanol + 2 CO_2	0.90
Glucose + 2 H_2O → 2 Glycerol + O_2	0.004
Glucose + 2 CO_2 → 2 Succinic acid + O_2	0.006
Glucose → 3 Acetic acid	0.015
Glucose → 2 Lactic acid	0.002
3 Xylose→ 5 Ethanol + 5 CO_2	0.85
3 Xylose + 5 H_2O → 5 Glycerol + 2.5 O_2	0.003
3 Xylose + 5 CO_2 → 5 Succinic acid + 2.5 O_2	0.009
2 Xylose → 5 Acetic acid	0.014
3 Xylose → 5 Lactic acid	0.002
Xylose + H_2O → Xylitol + 0.5 O_2	0.0046

Table S6 Market prices of ethanol and its co-products used for economic allocation of environmental impacts

Product	Price (2012)[a]	Source
Ethanol	808 £/t	www.icis.com/energy/ethanol
Acetic acid	407 £/t	www.icis.com/chemicals/acetic-acid
Lactic acid	1027 £/t	www.nnfcc.co.uk/publications/nnfcc-renewable-chemicals-factsheet-lactic-acid
Electricity	69 £/MWh	[8]

[a] Original prices adapted for inflation and currency exchange rate in 2012.

References

[1] Kurian, J.K., Nair, G. R., Hussain, A., Raghavan, V., Feedstocks, logistics and pre-treatment processes for sustainable lignocellulosic biorefineries: A comprehensive review. *Renewable and Sustainable Energy Reviews* 2013; 25 205–219.

[2] IEA. Sustainable production of second-generation biofuels - Potential and perspectives in major economies and developing countries. International Energy Agency, Paris. 2010. www.iea.org/papers/2010/second_generation_biofuels.pdf.

[3] Cherubini, F., and Ulgiati, S. Crop residues as raw materials for biorefinery systems - A LCA case study. *Applied Energy* 2009; 87(1) 47-57.

[4] Wooley, R., Ruth, M., Sheehan, J. and Ibsen, K. Lignocellulosic Biomass to Ethanol Process Design and Economics Utilizing Co-Current Dilute Acid Prehydrolysis and Enzymatic Hydrolysis Current and Futuristic Scenarios. 1999, NREL, Colorado.

[5] Phillips S, Aden A, Jechura J, and Dayton D, Thermochemical Ethanol via Indirect Gasification and Mixed Alcohol Synthesis of Lignocellulosic Biomass. United States, 2007.

[6] De Vrije, T., De Haas, G. G., Tan, G. B., Keijsers, E. R. P. Claassen, P. A. M., Pretreatment of miscanthus for hydrogen production by Thermotoga elfii. *Int J of Hydrogen Energy* 2002; 27(11) 1381-1390.

[7] Vassilev, S. V., Baxter, D., Andersen, L. K., and Vassileva, C. G. An overview of the chemical composition of biomass. *Fuel* 2010; 89(5) 913-933.

[8] UK Government. Climate change and sustainable energy act 2006. London: HMSO; 2006.
